# Temperature Sensing in the Short-Wave Infrared Spectral Region Using Core-Shell NaGdF_4_:Yb^3+^, Ho^3+^, Er^3+^@NaYF_4_ Nanothermometers

**DOI:** 10.3390/nano10101992

**Published:** 2020-10-09

**Authors:** Daria Pominova, Vera Proydakova, Igor Romanishkin, Anastasia Ryabova, Sergei Kuznetsov, Oleg Uvarov, Pavel Fedorov, Victor Loschenov

**Affiliations:** Prokhorov General Physics Institute of the Russian Academy of Sciences, Moscow 119991, Russia; vera.proydakova@gmail.com (V.P.); igor.romanishkin@nsc.gpi.ru (I.R.); nastya.ryabova@nsc.gpi.ru (A.R.); ksv@lst.gpi.ru (S.K.); uvarov@kapella.gpi.ru (O.U.); ppff@lst.gpi.ru (P.F.); loschenov@nsc.gpi.ru (V.L.)

**Keywords:** thermometry, luminescence, short-wave infrared, rare-earth ions

## Abstract

The short-wave infrared region (SWIR) is promising for deep-tissue visualization and temperature sensing due to higher penetration depth and reduced scattering of radiation. However, the strong quenching of luminescence in biological media and low thermal sensitivity of nanothermometers in this region are major drawbacks that limit their practical application. Nanoparticles doped with rare-earth ions are widely used as thermal sensors operating in the SWIR region through the luminescence intensity ratio (LIR) approach. In this study, the effect of the shell on the sensitivity of temperature determination using NaGdF_4_ nanoparticles doped with rare-earth ions (REI) Yb^3+^, Ho^3+^, and Er^3+^ coated with an inert NaYF_4_ shell was investigated. We found that coating the nanoparticles with a shell significantly increases the intensity of luminescence in the SWIR range, prevents water from quenching luminescence, and decreases the temperature of laser-induced heating. Thermometry in the SWIR spectral region was demonstrated using synthesized nanoparticles in dry powder and in water. The core-shell nanoparticles obtained had intense luminescence and made it possible to determine temperatures in the range of 20–40 °C. The relative thermal sensitivity of core-shell NPs was 0.68% °C^−1^ in water and 4.2% °C^−1^ in dry powder.

## 1. Introduction

The development of contactless thermal sensors has attracted much attention in the last decade [1,2,3,4]. Conventional thermocouples and thermometers are unsuitable for remote temperature control [5]. Precise contactless monitoring and temperature control at the microscopic level are highly important for controlling various biological functions and their changes during thermal therapies as well as visualization of thermal changes in vivo [6,7,8,9,10,11]. Contactless luminescent nanothermometers are promising for biomedical applications. Various luminescent materials with temperature-dependent optical features have been studied as optical thermometers. The most popular are quantum dots [12,13], organic molecules [14,15,16], polymers [17,18,19], DNA or protein-conjugated systems [20], and lanthanide-doped nanophosphors [21,22].

The depth of light penetration limits the use of optical thermometry methods for biological tissues [23,24]. Obtaining diagnostic information from deep layers of biological tissues such as the brain or the skin is complicated due to their heterogeneity and scattering [25,26]. Recent studies have shown that the low scattering in the short-wave infrared region (SWIR, 1100–2000 nm, the so-called second and third biological tissue transparency window) significantly increases the depth and sensitivity of fluorescence imaging, and provides excellent contrast and high signal-to-noise ratio for the obtained luminescent images [27,28,29].

However, the practical application of SWIR imaging and thermometry is still limited by a small number of SWIR phosphors. Most organic SWIR dyes and many types of nanomaterials developed for lasers and the telecommunications industry are unsuitable because of their low quantum efficiency (<0.1%), poor solubility in water, and potential toxicity. The organic dyes known to be fluorescent in the SWIR region, such as IR-26, IR-1048, and IR-1061, are highly hydrophobic, have an extremely low quantum yield (less than 0.05%), and contain toxic metals [30,31]. In the last decade, much attention has been paid to several classes of dyes and nanomaterials: cyanine dyes, lanthanide complexes, platinum complexes, certain types of quantum dots, small gold nanoparticles, and single-walled carbon nanotubes [32]. Inorganic nanoparticles containing rare-earth ions (REIs) may also be of interest. The advantages of rare-earth ions as luminescent labels include narrow-band radiation, a large spectral distance between excitation and emission wavelengths (which is characteristic for the up- and downconversion), long luminescence lifetime, high photostability and low toxicity of materials, minimal autofluorescence of biological tissues, and the greatest depth of sounding when excited in the near-infrared region (NIR) [33]. In comparison with other phosphors, rare-earth ions can be excited through multiple electron states and, due to internal conversion, can produce luminescence bands in a wide range of visible, NIR, and SWIR spectra [34].

Obtaining SWIR-based luminescent markers with high quantum efficiency under infrared excitation is an urgent task today. This study was devoted to the synthesis of NaGdF_4_ nanoparticles doped with rare-earth ions Yb^3+^, Ho^3+^, and Er^3+^ covered with an inert NaYF_4_ shell, and to the study of the effect of the shell on the characteristics of luminescence and thermal sensitivity of the synthesized nanoparticles at various pump power densities.

The luminescence intensity ratio approach, which tracks temperature-induced changes in luminescence intensity at two different wavelengths, is widely used for thermal sensing [35,36]. This self-referential technique allows the dependency on measurement conditions to be reduced and high sensing accuracy to be achieved.

Two main approaches can be used for temperature sensing: the luminescence intensity ratio from a single type of lanthanide ions or from two or more types doped into a single host matrix. To quantitatively assess the thermometric capability of a nanothermometer, the sensor sensitivity is usually used, defined as the rate of change of the temperature-sensitive parameter with temperature. The first single-ion approach is based on the temperature-dependent emission from two thermally coupled levels, and is therefore limited by the energy gap between them. In the second approach, the temperature-induced emission changes from the luminescence intensity ratio of two or more different lanthanide ions doped into a single host matrix are analyzed, wherein only one emission is influenced by temperature changes depending on the surrounding medium.

The majority of lanthanide-based nanothermometers in the visible (VIS) or NIR region operate via the upconversion luminescence intensity ratio approach [37]. To overcome the limitations attributed to low light penetration depth in this region, developing downconversion nanoparticles with the excitation and emission wavelength matching one of the three biological windows has been suggested [38,39,40]. In those regions, the tissue becomes partly transparent, allowing for higher penetration depth, better spatial resolution, and reduced side effects [41]. One study demonstrated the fundamental possibility of contactless thermometry in the SWIR spectral range using nanoparticles doped with rare-earth ions [42]. The same group also optimized the concentration of doped impurities of triply doped NaYF_4_: Yb^3+^, Ho^3+^, Er^3+^ core-only nanoparticles (NPs) operating in the over-1000 nm region to obtain high sensitivity to temperature changes [43]. The maximum sensitivity was 2.15% °C^−1^ in water and 2.17% °C^−1^ in cyclohexane.

The synthesis of core-shell LaF_3_ NPs for bioimaging in SWIR was reported recently [44]. The relative thermal sensitivity obtained ranged from 0.1% °C^−1^ (core only) to 0.41% °C^−1^ and 0.36% °C^−1^ (core-shell), but was lower compared to their upconversion counterparts [45,46]. The relative sensitivity values of 1.1% °C^−1^ were obtained for NaGdF_4_: Er^3+^, Ho^3+^, Yb^3+^, Nd^3+^ core-multi-shell NPs operating in the NIR [47]. However, core-multi-shell materials are usually complex (multilayer structure) and their synthesis is a multistep process. In another study, the use of the Yb^3+^/Er^3+^ 1010/810 and 1010/660 nm emission band ratios of core-shell β-NaYF_4_:Yb^3+^−Er^3+^@SiO_2_ nanorods for temperature-sensing purposes was presented and a sensitivity of 1.64% °C^−1^ was obtained for 1010/810 nm luminescence intensity ratios [48].

Several groups have studied the dependence of temperature determination sensitivity on structural properties of NPs such as composition, phonon energy of the host material [49], NP size [50], and the dopant concentration [51], as well as on the influence of the surrounding medium [38,52]. In water, the overall emission intensity is lower due to an increase in water-induced non-radiative losses. These mainly affect the sensitizer Yb^3+^ [53,54] and therefore suppress the activator emission. It has previously been shown that the quenching of luminescence in the VIS and NIR regions could be prevented by using core-shell or complex core-multi-shell structures [55,56,57]. In this study, we examined the influence of NaYF_4_ inert shell on the luminescence intensity in SWIR and the thermal sensing sensitivity of Yb^3+^, Ho^3+^, Er^3+^ tri-doped NaGdF_4_ NPs. We also investigated the effect of the pump power density on laser-induced heating and thermal sensitivity. High pump power densities were shown to cause intense heating, which negatively affected the thermometric properties of the studied nanoparticles. At low pump power densities, the luminescence intensity was low and strongly quenched in water, which negatively affected the accuracy of temperature determination. Coating nanoparticles with an inert shell can significantly improve luminescence characteristics, prevent quenching, and obtain high thermal sensitivity.

## 2. Materials and Methods

The NaGdF_4_ NPs doped with Yb^3+^, Ho^3+^, and Er^3+^ ions was synthesized via the solvothermal technique in oleic acid [58]. The concentration ratio for ions was 20:3:0.5, previously described as optimal for thermosensing in SWIR [43]. Rare-earth element acetates (99.99% purity, Lanhit, Moscow, Russia), sodium hydroxide (NaOH) and ammonium fluoride (NH_4_F) with chemical purity (Lanhit, Moscow, Russia), oleic acid (Sigma Aldrich, St. Louis, MO, USA), and 1-octadecene (Sigma Aldrich, St. Louis, MO, USA) were used as precursors. Ytterbium, erbium, holmium, and gadolinium acetates; oleic acid; and 1-octadecene were placed in a 250 mL three-necked flask under reflux. The reaction mixture was heated to 140 °C under argon with vigorous stirring until the acetates were completely dissolved and transformed into rare-earth oleates. Water and acetic acid were then removed under vacuum and the reaction mixture was cooled down to room temperature. The appropriate solutions of NaOH and NH_4_F in methanol were added in stoichiometric amounts with respect to rare-earth acetates. The reaction mixture was then heated up to 50–60 °C and held for one hour under vacuum to remove the methanol and cause the nucleation of cubic phase α-NaGdF_4_:Yb^3+^, Ho^3+^, Er^3+^ NPs. After the removal of the methanol, the reaction mixture was heated up to a predetermined temperature of 295 °C. The temperature was maintained for 1.5–2 h to transition from cubic to the hexagonal phase β-NaGdF_4_:Yb^3+^, Ho^3+^, Er^3+^ and then cooled to 25 °C.

The synthesis of NaYF_4_ shell involved the same steps. Yttrium acetate, oleic acid, and 1-octadecene were added to the three-necked flask under reflux. The reaction mixture was then heated up to 140 °C in argon with vigorous stirring. Water and acetic acid were removed under vacuum and the reaction mixture was cooled down to room temperature. After heating to 50–60 °C and the removal of methanol, the reaction mixture was heated to 295 °C and held for 1.5–2 h for NaYF4 formation, then cooled to 25 °C. The NPs were collected by centrifugation (8500 rpm, 5 min). The resulting NPs were washed three times with chloroform and ethanol. The determination of the phase composition of the synthesized samples was performed via the X-ray powder diffraction (XRD) technique (D8 Bruker^®^ Advance diffractometer with Cu-Kα radiation, Powder 2.0 software from Laboratory of Inorganic Crystallochemistry, MSU by Oleynikov Peter, Moscow, Russia). The particle size and morphology were analyzed using transmission electron microscopy (TEM, Libra 200 FE microscope, Carl Zeiss AG, Oberkochen, Germany) with ImageJ software (version 1.53a, open source Java image processing program, https://imagej.net).

For the temperature-dependent luminescence spectra measurements, the samples were spread thinly over a horizontal aluminum plate and excited using a semiconductor laser with a wavelength of 980 nm, which was operated in a continuous wave regime. A dry sample was also suspended in water to study its influence on SWIR luminescence. A thin layer of the colloid (1 mm) was poured into an open cuvette. The laser beam was focused at a spot 1 cm in diameter with pump power density in the 0.4–4.0 W/cm^2^ range. The luminescence spectra in the 1000–1700 nm region were measured using an optical fiber spectrometer DWARF-Star (StellarNet, Tampa, Fl, USA). A hard-coated edge pass filter FELH-1050 (Thorlabs, Newton, N.J., USA) was used to filter the signal from the laser excitation. The luminescence was captured with a fiber with a core diameter of 200 μm. Temperature was controlled using thermometric infrared camera JADE MWIR SC7300M (CEDIP, Croissy-Beaubourg, France) synchronized with the spectrometer for the simultaneous measurement of the luminescence spectrum and the sample temperature.

The temperature-dependent emission spectra of the NaGdF_4_:Yb^3+^, Ho^3+^, Er^3+^ and NaGdF_4_:Yb^3+^, Ho^3+^, Er^3+^@NaYF_4_ NPs were analyzed. To quantify the thermal sensitivity, the ratio between the integral intensities of Ho^3+^ in the 1125–1200 nm range (I_Ho_) and Er^3+^ in the 1450–1650 nm range (I_Er_) emission peaks (luminescence intensity ratio, LIR) were used:

LIR = I_Ho_/I_Er_. (1)

The relative thermal sensitivity of the NaGdF_4_:Yb^3+^, Ho^3+^, Er^3+^ and NaGdF_4_:Yb^3+^, Ho^3+^, Er^3+^@NaYF_4_ NPs was determined by the ratio [59]:

S = 1/LIR × dLIR/dT.(2)

## 3. Results and Discussion

**Figure 1:**
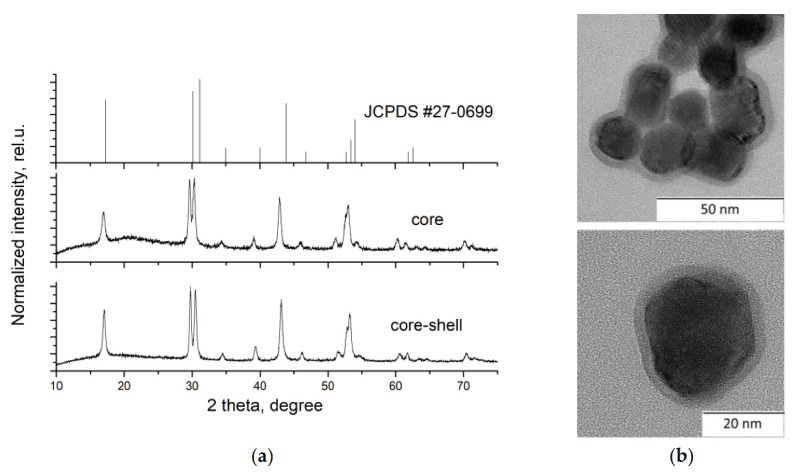
(**a**) XRD diagrams of JCPDS #27-0699 and NaGdF_4_:Yb^3+^, Ho^3+^, Er^3+^ core and core-shell nanoparticles and (**b**) TEM images of NaGdF_4_:Yb^3+^, Ho^3+^, Er^3+^@NaYF_4_ core-shell nanoparticles.

As a result, the NaGdF_4_:Yb^3+^, Ho^3+^, Er^3+^, and NaGdF_4_:Yb^3+^, Ho^3+^, Er^3+^@NaYF_4_ NPs (Yb^3+^:Ho^3+^:Er^3+^ = 20:3:0.5 mol %) were synthesized. The notations “core” and “core-shell” are used hereafter, respectively. According to XRD data from JCPDS #27-0699, the synthesized samples were a pure hexagonal phase. The X-ray powder patterns of the obtained samples are presented in Figure 1.

The deviation of the X-ray peak positions was associated with the difference in the radii of the gadolinium, ytterbium, erbium, holmium, and yttrium cations [60], which resulted in the unit cell parameters changing from *a* = 6.020 Å, *c* = 3.601 Å for JCPDS #27-0699 to *a* = 6.028(2) Å, *c* = 3.569 Å for the core and *a* = 6.006(1) Å, *c* = 3.549(1) Å for the core-shell NPs. The NaGdF_4_:Yb^3+^, Ho^3+^, Er^3+^@NaYF_4_ sample consisted of particles with obvious core-shell architecture with semi-spherical morphology and an average diameter of 23 nm (Figure 1b). The thickness of the shell was about 3 nm.

**Figure 2:**
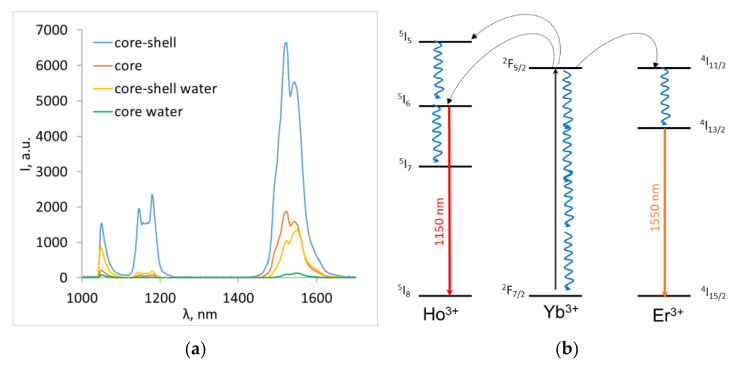
(**a**) The luminescence spectra of NaGdF_4_:Yb^3+^, Ho^3+^, Er^3+^ core and core-shell nanoparticles measured at 30 °C in powder and in aqueous solution and (**b**) simplified energy level diagram of the Yb^3+^-, Ho^3+^-, and Er^3+^-emitting centers.

The luminescence spectra of the obtained core and core-shell nanoparticles in powder and in water are shown in Figure 2.

All samples emitted the characteristic Ho^3+^ and Er^3+^ luminescence at 1150 nm (^5^I_6_ → ^5^I_8_) and 1550 nm (^4^I_13/2_ → ^4^I_15/2_) with no evident change in the band position. The emission intensity was highly dependent on the interaction with the surrounding medium and decreased in water. The decrease was more pronounced for the core nanoparticles: the integral luminescence intensity in 1050–1600 nm region decreased 5-fold for core-shell NPs compared to 14-fold for core-only NPs. This phenomenon is well known and could be explained by the quenching of surface-ion luminescence by high vibrational energy ligands in the surrounding medium or surface defects [61]. The non-radiative losses mainly affected the sensitizer (Yb^3+^) and therefore suppressed the Ho^3+^ and Er^3+^ emission.

**Figure 3:**
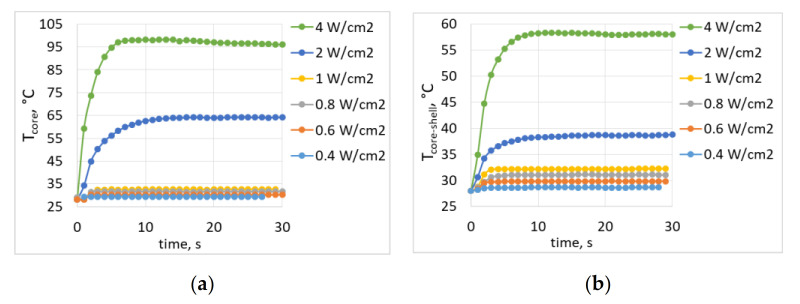
The influence of pump power density on heating temperature of (**a**) core NaGdF_4_:Yb^3+^, Ho^3+^, Er^3+^ NPs and (**b**) core-shell NaGdF_4_:Yb^3+^, Ho^3+^, Er^3+^ NPs.

A study of laser-induced heating of core and core-shell particles was conducted by varying laser power density between 0.4 and 4.0 W/cm^2^ (Figure 3).

We noted that the core NPs heated up much more, especially at high pump power densities. After 10 s of irradiation with 2 W/cm^2^ pump power density, the temperature reached 38 °C for core-shell and 64 °C for core NPs. The differences were less significant at lower pump power densities, and the heating temperature was about 29 °C at 0.4 W/cm^2^ (the lowest power density used) and 32.5 °C at 1 W/cm^2^ both for the core and the core-shell NPs. In this case, heating to an equilibrium temperature occurred in less than 10 s. For further spectroscopic studies of the dependence of the luminescence spectra on the power density during laser-induced heating, the temperature in the time range of 10–30 s (when the temperature was already stable) was averaged.

**Figure 4:**
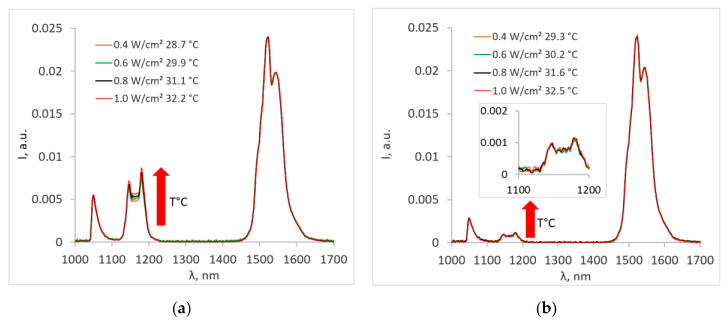
The influence of pump power density and, consequently, the temperature on the emission spectra of nanoparticles in powder: (**a**) core-shell and (**b**) core.

The influences of pump power density and, consequently, the temperature on the emission spectra of NPs in powder are shown in Figure 4a.

It was evident that for the dry sample, the Ho^3+^ emission rose with the increase in temperature for core-shell nanoparticles. This dependence could be explained by the non-resonant nature of the Yb^3+^ → Ho^3+^ transition. In this case, the energy transfer is facilitated by the phonon-assisted processes, where the energy mismatch is compensated by simultaneous emission or absorption of one or more phonons by the host lattice [62]. Because the phonon-assisted energy transfer is temperature-dependent [63], the Ho^3+^ emission tends to increase with the increased temperature. The Yb^3+^ → Er^3+^ energy transfer is resonant, so the temperature change does not affect the Er^3+^ emission. It was also noted that the temperature-dependent changes in the luminescence spectrum of the core nanoparticles (Figure 4b) were negligible and difficult to detect due to the low intensity of the characteristic Ho^3+^ peak at a wavelength of 1150 nm.

**Figure 5:**
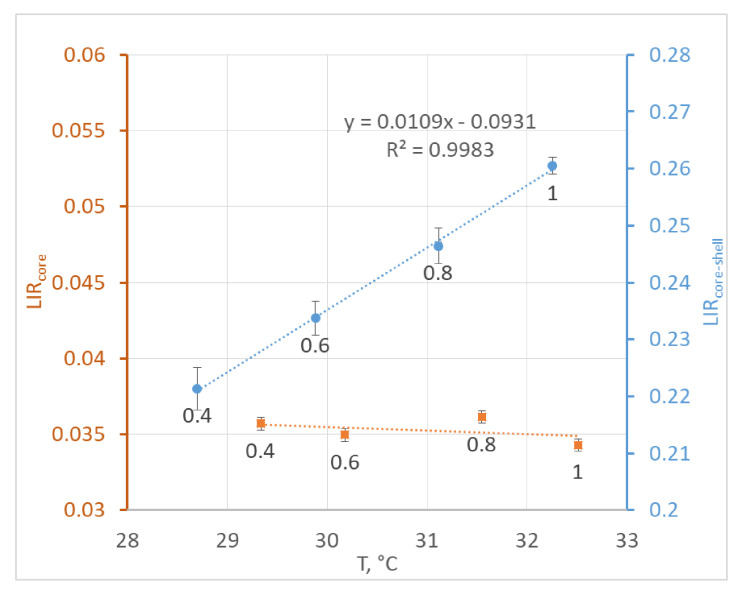
The dependence of luminescence intensity ratio (LIR) on the temperature for the core and core-shell nanoparticles in powder. The pump power density in W/cm^2^ is shown as data labels.

The LIR values, which were determined from the temperature-dependent emission spectra, are presented in Figure 5.

The observed dependency of LIR on temperature for the core-shell NPs was almost linear, whereas for the core NPs, the observed changes in LIR were within the measurement error. The relative thermal sensitivity for core-shell nanoparticles in powder was determined to be 4.2% °C^−1^.

**Figure 6:**
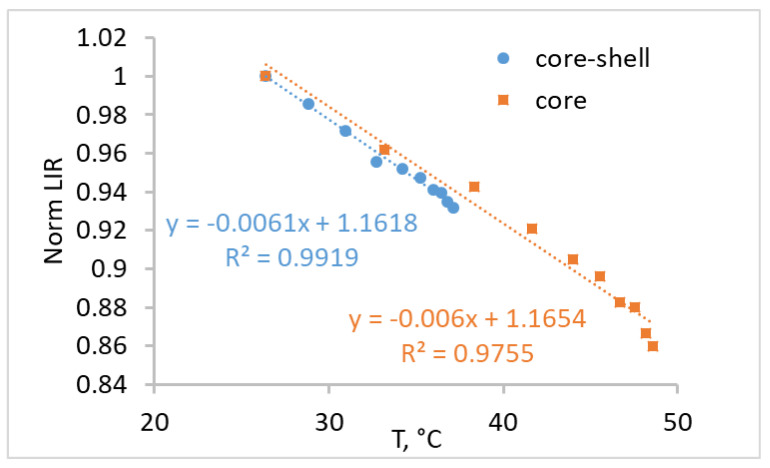
The dependence of luminescence intensity ratio (LIR) normalized to the value at 29 °C on the heating temperature for the core and core-shell nanoparticles in water.

Laser-induced heating of synthesized core and core-shell nanoparticles in water was also studied. As with dry powder, the heating temperature of the core NPs was higher than for core-shell NPs, amounting to 48 °C for the core and 37 °C for the core-shell NPs at 1 W/cm^2^. The dependence of LIR on the heating temperature for the core and core-shell NPs in water is presented in Figure 6.

The dependence of LIR on temperature is significantly different in water than in dry powder. Another slope of dependence was attributed to the interaction with the environment [38,39]. The O‒H vibrational stretching modes of water affected the relaxation processes of the ^5^I_6_ →^5^I_7_, ^5^I_5_→^5^I_6_ transitions of Ho^3+^, and the ^4^I_11/2_ → ^4^I_13/2_ transition of Er^3+^ (Figure 2b). This led to an almost constant intensity of Ho^3+^ emission attributed to the ^5^I_6_ →^5^I_8_ transition. At the same time, the Er^3+^ emission was raised by enhanced ^4^I_11/2_ →^4^I_13/2_ vibrational relaxation, which led to an increase in the population emitting the ^4^I_13/2_ level. The influence of vibrational relaxation increased with the temperature.

Coating particles with an inert shell had no significant effect on the character and slope of the obtained dependence of LIR on the temperature (Figure 6). This was attributed to Yb^3+^ ions interacting more strongly with vibrational modes of water than the Er^3+^ and Ho^3+^ ions [61]. It is well known that Yb–Yb energy migration is efficient and can travel long distances until it is quenched by the surface [64]. Using an inert shell is therefore an effective strategy to prevent quenching and enhance brightness. The relative thermal sensitivity in the 26–36 °C region for the core and core-shell nanoparticles in water was determined to be 0.67 and 0.68% °C^−1^, respectively.

The detected luminescence signal was significantly lower for uncoated particles than for coated particles. Additionally, a significant decrease in the luminescent signal intensity for uncoated particles was observed in biological media, both due to the quenching of the Yb^3+^ donor and the quenching of the luminescence of Er^3+^ and Ho^3+^. This resulted in the need for high pump power density to obtain a sufficient signal intensity. High pump power can induce the heating of the particles, which could reduce the sensitivity and accuracy of temperature determination and also lead to damage to biological samples. Coating nanoparticles with an inert shell could significantly increase the signal intensity and reduce the measurement error. At the same time, the positive effect of the shell occurs mainly in the reduction of Yb^3+^ quenching and not in the higher sensitivity of thermometry by the luminescence of Er^3+^ and Ho^3+^.

## 4. Conclusions

Tri-doped Yb^3+^, Ho^3+^, Er^3+^ core and core-shell β-NaGdF_4_ NPs were successfully synthesized in this study.

The influence of NaYF_4_ inert shell on the intensity of luminescence in SWIR and the thermal sensing sensitivity of Yb^3+^, Ho^3+^, Er^3+^ tri-doped NaGdF_4_ NPs was investigated. We found that in the case of core NPs, higher pump power density is needed for Ho^3+^ excitation and the detection of temperature-dependent changes in the Ho^3+^ luminescence spectrum. We also demonstrated that an increase in pump power density leads to laser-induced heating, which is stronger in the case of core NPs and could negatively affect the thermometric properties of the NPs and lead to degradation of samples. Coating nanoparticles with an inert shell can significantly improve the characteristics of luminescence, prevent quenching, and obtain high thermal sensitivity. The relative thermal sensitivity for core-shell nanoparticles in dry powder was determined to be 4.2% °C^−1^.

Coating the NPs with an inert shell was also shown to prevent the quenching of SWIR luminescence by water. The decrease in integral luminescence intensity in the 1050–1600 nm region in water was 5-fold for the core-shell NPs and 14-fold for the core-only NPs. The coating of the particles with an inert shell had no significant effect on the character and slope of the obtained dependence of LIR on the temperature. We attribute this to Yb^3+^ ions interacting more strongly with vibrational modes of water than Er^3+^ and Ho^3+^ ions. The relative thermal sensitivity in the 26–36 °C region in water for the core and core-shell nanoparticles was determined to be 0.67 and 0.68% °C^−1^, respectively.

Thus, core-shell nanothermometers demonstrated a high intensity of the luminescent signal in SWIR and high thermal sensitivity in both water (0.68% °C^−1^) and dry powder (4.2% °C^−1^).
